# *Allium hookeri* extracts inhibit cisplatin-induced apoptosis and inflammation in human kidney HEK-293 cells

**DOI:** 10.29219/fnr.v69.10764

**Published:** 2025-06-05

**Authors:** Ha-Rin Moon, Wooje Lee, Jung-Mi Yun

**Affiliations:** 1 Department of Food and Nutrition, Chonnam National University, Yongbong-ro, Buk-gu, Gwangju, South Korea; 2 Technology Innovation Research Division, World Institute of Kimchi, 86 Kimchi-ro, Nam-gu, Gwangju, Republic of Korea

**Keywords:** *Allium hookeri*, cisplatin, apoptosis, inflammation, HEK-293

## Abstract

**Background:**

Cisplatin is widely utilized in the treatment of solid malignant tumors due to its potent anticancer effects through the inhibition of cell division. However, its clinical use is often limited by significant adverse effects, particularly nephrotoxicity. Recent research has focused on natural products as potential mitigators of cisplatin-induced kidney toxicity. *Allium hookeri* (*A. hookeri*), a traditional food and herbal medicine in Southeast Asia, is known for its antioxidant and anti-inflammatory properties. However, its protective effects against nephrotoxicity remain unclear.

**Objective:**

This study aimed to investigate the protective effects of *A. hookeri* against cisplatin-induced nephrotoxicity in human embryonic kidney (HEK)-293 cells.

**Methods:**

HEK-293 cells were treated with cisplatin (50 μM) with or without *A. hookeri* water extract (AHWE) and ethanol extract (AHEE) for 24 h. Cell viability was assessed using MTT assays, and nuclear morphology was examined through Hoechst 33342 staining. Intracellular reactive oxygen species (ROS) production was quantified using ROS detection assays, and nitric oxide (NO) production was measured through Griess reaction assays. Protein and mRNA expression levels were analyzed using western blotting and quantitative polymerase chain reaction (qPCR) techniques.

**Results:**

Cisplatin treatment (50 μM) significantly increased ROS production compared to untreated cells within 24 h. Both AHWE and AHEE treatments markedly attenuated ROS generation. Additionally, AHWE and AHEE significantly inhibited NO production and downregulated the expression of inflammation-related genes. The treatments also suppressed mitogen-activated protein kinase (MAPK) protein expression. Pretreatment with AHWE and AHEE decreased the Bax/Bcl-2 expression ratio, demonstrating a dose-dependent inhibition of apoptotic features.

**Conclusion:**

The findings suggest that *A. hookeri* exerts protective effects against cisplatin-induced kidney damage by modulating MAPK signaling, thereby reducing inflammation and apoptosis in HEK-293 cells. *A. hookeri* represents a promising therapeutic candidate for the prevention and treatment of nephrotoxicity.

## Popular scientific summary

- Allium Hookeri extract suppress apoptosis by modulating the Bax to Bcl-2 ratio in cisplatin-induced nephrotoxic HEK-293 cells.
- Allium Hookeri extract inhibit cell apoptosis by suppressing the MAPK signaling pathway in cisplatin-induced nephrotoxic HEK-293 cells.
- Allium Hookeri extract significantly attenuated intracellular ROS generation in cisplatin-induced nephrotoxic HEK-293 cells.
- Allium Hookeri extract suppressed cisplatin-induced nitric oxide production and the expression of inflammation-related genes (TNF-α and IL-6) and NF-κB target genes in cisplatin-induced nephrotoxic HEK-293 cells.

The kidney plays a critical role in maintaining physiological homeostasis by regulating fluid balance, maintaining acid-base equilibrium, excreting waste products, and functioning as an endocrine organ (1, 2). Nephrotoxicity, a frequent and serious complication, can be triggered by a range of pharmacological agents, including cisplatin, aminoglycosides, and antiretroviral drugs (3).

Cisplatin, an inorganic platinum-based heavy metal complex, is widely used in chemotherapy for the treatment of solid organ malignancies, including testicular, ovarian, and cervical cancers, due to its potent antitumor efficacy (4). However, its clinical utility is limited by significant adverse effects, including emesis, bone marrow suppression, neurotoxicity, and, notably, nephrotoxicity (5). Emerging research using cellular and animal models of cisplatin-induced nephrotoxicity has underscored the pivotal roles of inflammation, oxidative stress, and cell death in this condition (6, 7). Cisplatin administration is known to provoke the generation of reactive oxygen species (ROS), including hydroxyl radicals, primarily through mitochondrial dysfunction. This cascade further initiates apoptotic pathways involving the activation of caspases and the mitogen-activated protein kinase (MAPK) pathway as inflammatory mediators (8). Nevertheless, the precise signaling mechanisms underlying cisplatin-induced kidney injury remain inadequately elucidated.

Current investigations are focused on the potential of natural compounds derived from safe and diverse dietary sources to prevent and treat cisplatin-induced nephrotoxicity (9). Notably, phytochemicals such as quercetin, dioscin, and vanillin have demonstrated efficacy in mitigating renal injury (10–12). *Allium hookeri* (*A. hookeri*), a member of the Liliaceae family predominantly found in East Asia, contains sulfur compounds, including S-allylcysteine (13). This plant exhibits a broad spectrum of physiological activities, including antidiabetic, antidementia, and antibacterial properties (14–16). Traditionally, *A. hookeri* has been widely utilized in traditional medicine and as a culinary ingredient in Asian cuisine. However, its potential protective effects against cisplatin-induced nephrotoxicity remain unexplored.

Oxidative stress induced by excessive ROS production is a well-recognized contributor to the pathogenesis of cisplatin-induced nephrotoxicity (8). Elevated ROS levels enhance the expression of pro-inflammatory mediators such as inducible nitric oxide synthase (iNOS), nuclear factor kappa B (NF-κB), and tumor necrosis factor-α (TNF-α) (17). These processes activate apoptotic signaling pathways through the upregulation of pro-inflammatory cytokines and the impairment of immune cell function (18). Clinical studies have shown elevated levels of inflammatory cytokines in patients with kidney injury (19). Preclinical research by Faubel et al. demonstrated increased serum concentrations of interleukin (IL)-6 and TNF-α in a mouse model of cisplatin-induced nephrotoxicity (20). Furthermore, Wang et al. reported that isoquercitrin alleviated nephrotoxicity by suppressing the expression of TNF-α, IL-6, and cyclooxygenase-2 (COX-2) in cisplatin-treated HK2 cells (21).

Recent clinical data indicate that renal cell death occurs in 20–30% of patients with acute kidney injury following cisplatin chemotherapy (22). Mechanistically, cisplatin-induced ROS generation facilitates the translocation of activated Bax to the outer mitochondrial membrane, initiating apoptotic cell death (23, 24). The antiapoptotic protein Bcl-2 counteracts this process by inhibiting cell apoptosis during renal injury (25).

The MAPK signaling pathway plays a crucial role in regulating apoptosis and other cellular processes, including cell transformation and proliferation (26). Cellular stress induced by cisplatin activates MAPK pathways, such as extracellular signal-regulated kinases (ERK), c-Jun N-terminal kinases (JNK), and p38 MAPK (p38) (27, 28). Suppression of ERK, p38, or JNK expression has been shown to reduce inflammation, caspase activation, apoptotic cell death, and renal damage (29).

Therefore, this study aims to investigate the renal protective effects and elucidate the intracellular mechanisms of *A. hookeri* in cisplatin-induced nephrotoxicity using human embryonic kidney (HEK)-293 cells.

## Materials and methods

### Materials

HEK-293 cells were obtained from the Korean Cell Line Bank (Seoul, South Korea). Cisplatin was purchased from Fujifilm Wako Pure Chemical Corporation (Osaka, Japan). *A. hookeri* root dry powder was procured from an online open market (Gmarket, Seoul, Korea). 3-(4,5-Dimethylthiazol-2-yl)-2,5-diphenyltetrazolium bromide (MTT), sulfanilamide, naphthyl ethylenediamine dihydrochloride, and acetic acid were acquired from Sigma Aldrich (St. Louis, MO, USA). Quantitative polymerase chain reaction (qPCR) primers (Bax, Bcl-2, NF-κB, TNF-α, COX-2, and β-actin) were sourced from Bioneer (Daejeon, South Korea). The Bcl-2 was purchased from Abcam (Cambridge, UK), while Bax, p-ERK, p-38, TNF-α, COX-2, and NF-κB antibodies were obtained from Santa Cruz Biotechnology (Santa Cruz, CA, USA). p-ERK and secondary antibody were provided by Cell Signaling Technology (Beverly, MA, USA). The BCA protein assay kit was provided by Thermo Fisher Scientific (Waltham, MA, USA). Unless otherwise stated, all extraction solvents and other chemicals were sourced from Sigma Aldrich or Biosesang (Sungnam, Gyeonggi-do, Korea).

### Preparation of the A. hookeri extract

The *A. hookeri* root hot water extract (AHWE) was prepared by adding 10 times the volume of water per gram of dry powder. The mixture was stirred under reflux cooling at 95°C for 4 h and subsequently filtered. The 80% ethanol extract (AHEE) was produced using the same water-to-powder ratio, stirred under reflux cooling at 95°C for 8 h, and then filtered. The extracts were concentrated under reduced pressure using a rotary vacuum evaporator (EYELA N-1000, Tokyo, Japan) and dried to determine the solid content. The yields of AHWE and AHEE were calculated to be 34.25 and 22.45%, respectively. The extracts were stored at -80°C until further experimental analysis.

### AHWE, AHEE, and cisplatin treatment in HEK-293 cells

HEK-293 cells were cultured in Dulbecco’s modified Eagle’s medium supplemented with 10% (v/v) fetal bovine serum and 1% penicillin/streptomycin (Welgene, Daegu, Korea) and maintained at 37°C in a humidified 5% CO_2_ atmosphere. Cells were seeded at a density of 1 × 10^4^ cells/mL. Prior to exposure to 50-μM cisplatin for 24 h, cells were pretreated with AHWE and AHEE for 24 h.

### Measuring cell viability

Cell viability was assessed using an MTT assay to evaluate the cytotoxic effects of AHWE and AHEE on HEK-293 cells. After adding the MTT solution, cells were incubated for 2 h to facilitate formazan precipitation. The resulting formazan was dissolved in 1 mg/mL of 100% dimethyl sulfoxide and transferred to a plate. Absorbance at 570 nm was measured using a plate reader to quantify cell viability.

### Measuring nitric oxide production

Excessive nitric oxide (NO) production can lead to inflammation and cell death through oxidative stress. To evaluate the inhibitory effects of AHWE and AHEE on NO production induced by cisplatin, we employed the Griess reaction (30). Each cell-free culture supernatant was incubated with the same Griess reagent (1% sulfanilamide and 1% naphthyl ethylenediamine dihydrochloride in 30% acetic acid) for 15 min. Nitrite within the samples reacted with sulfanilamide and naphthyl ethylenediamine dihydrochloride to produce a pink azo dye. The absorbance of the resulting azo dye was measured at 562 nm using an EZRead 400 Microplate Reader (Biochrom, Cambridge, UK). A standard curve was generated using serial dilutions of sodium nitrite, which was subsequently applied to determine nitrite concentrations in the samples. Quantification of nitrite was calculated by plotting the average absorbance of each concentration on an X-Y plot, and the nitrite concentration using a linear regression curve. The experiment was conducted independently in triplicate.

### Measuring Intracellular ROS Levels

The impact of AHWE and AHEE on cisplatin-induced changes in intracellular ROS levels was evaluated using the DCFDA Cellular ROS Detection Assay Kit (Abcam, Cambridge, UK), according to the manufacturer’s protocol. The DCFDA assay is based on the conversion of DCF-DA to DCF upon reaction with intracellular ROS, enabling quantification of ROS generation through the concentration of converted DCF. Cells were incubated with DCFDA for 45 min at 37°C. ROS distribution was measured using a microplate fluorescence reader (Tecan, Zurich, Switzerland) at Ex/Em = 485/535 nm. The relative ROS level (%) was calculated using the following formula: Relative ROS level (%) = (Sample fluorescence intensity − Blank fluorescence intensity/Control fluorescence intensity) × 100. The experiment was independently repeated three times.

### Immunoblotting analysis

Immunoblotting analysis was performed to assess the expression levels of proteins associated with inflammation and apoptosis. Whole-cell lysates were prepared using RIPA buffer (Biosesang, Sungnam, Korea) supplemented with Halt™ protease and phosphatase inhibitor cocktail (Thermo Fisher Scientific, Waltham, MA, USA). Nuclear lysates were prepared using a nuclear extraction buffer containing 20 mM HEPES, 0.4 mM NaCl, 1 mM EDTA, 1 mM EGTA, 1 mM dithiothreitol, and 1 mM PMSF, 10% NP-40. Protein concentrations were measured using the BCA protein assay (Pierce, IL, USA) according to the manufacturer’s instructions. Proteins (20 μg) were separated using SDS-PAGE and subsequently transferred onto a nitrocellulose membrane (Invitrogen, Waltham, MA, USA). Membranes were blocked for 2 h in blocking buffer (10 mM Tris-HCl (pH 7.5), 150 mM NaCl, 0.1% Tween 20, and 5% non-fat dry milk) and then incubated with the appropriate primary antibodies for 2 h. Following primary antibody incubation and washing, membranes were incubated with a diluted conjugated secondary antibody for an additional 2 h. Blots were developed using the Western blotting luminol reagent (Santa Cruz Biotechnology, Dallas, TX, USA) and analyzed using the ChemiDoc XRS+ Imaging System (BioRad, Hercules, CA, USA). Protein expression levels were normalized to β-actin and quantified using ImageJ (a free online image analysis software). The following primary antibodies were used: Bcl-2 (abcam Cat#ab7973-1, 1:1000 dilution), Bax (Santa Cruz Cat#sc-7480, 1:1000 dilution), p-ERK (Cell Signaling Technology Cat# 9101s, 1:500 dilution), p-JNK (Cell Signaling Technology Cat#4668S, 1:1000 dilution), p-P38 (Santa Cruz Cat#sc-166182, 1:1000 dilution), TNF-α (Santa Cruz Cat#sc-133192, 1:1000 dilution), COX-2 (Santa Cruz Cat#sc-376861, 1:500 dilution), and NF-κB (Santa Cruz Cat#sc-8008, 1:1000 dilution). The secondary antibody used was Anti-rabbit IgG, HRP-linked Antibody (Cell Signaling Technology Cat# 7074P2, 1:2000 dilution), Anti-mouse IgG, HRP-linked Antibody (Cell Signaling Technology Cat# 7076P2, 1:2000 dilution).

### Quantitative real-time polymerase chain reaction analysis

Quantitative polymerase chain reaction was conducted to assess mRNA levels of genes involved in inflammation and apoptosis. Total RNA was extracted using Trizol reagent according to the protocol of the manufacturer (Thermo Fisher Scientific, Waltham, MA, USA). Total RNA concentration and purity were determined by measuring absorbance at 260 and 280 nm using a NanoDrop 2000 spectrophotometer (Thermo Fisher Scientific, Waltham, MA, USA). First-strand cDNA was synthesized from 1 μg of total RNA using the Omniscript RT kit (QIAGEN, Hilden, Germany). SYBR green-based quantitative PCR was conducted on a CFX96 Touch Real-Time PCR Detection System (BioRad, CA, USA), with all reactions performed in triplicate. Gene expression was quantified using the 2^-ΔΔCT^ method with β-actin as an internal control. The primer sequences used were as follows: human *bax*, forward 5′-TCCACCAAGAAGCTGAGCGAG-3′ and reverse 5′-GTCCAGCCCATGATGGTTCT-3′; *bcl-2*, forward 5′-TCCGCGTGATTGAAGACACC-3′ and reverse 5′-TCTCCCGGTTATCGTACCCT-3′; *NF-*κ*B*, forward 5′-GACAAGGTGCAGAAAGATGACAT-3′ and reverse 5′-TCATACGGTAACACAAGGCCT-3′; *TNF-*α, forward 5′-CAATGTAGGAGCTGCCTTGG-3′ and reverse 5′-CAGAGGCTCAGCAATGAGTG-3′; *COX-2*, forward 5′-AGATCATCTCTGCCTGAGTATCTT-3′ and reverse 5′-TTCAAATGAGATTGTGGGAAAATTGCT-3′; β*-actin*, forward 5′-CACCCCGTGCTGCTGAC-3′ and reverse 5′-CCAGAGGCGTACAGGGATAG-3′.

### Hoechst 33342 staining

Hoechst 33342 staining was utilized to evaluate nuclear condensation, a morphological change hallmark of cell death. Cells were fixed in 4% paraformaldehyde (PFA) for 30 min. Following fixation, the cells were washed with phosphate buffered saline (PBS) and then stained with Hoechst 33342 solution (10 μg/mL, Thermo Fisher Scientific, Waltham, MA, USA) for 10 min. A mounting solution was applied to a glass slide, and a coverslip was placed over it. Nuclear morphology was visualized using a fluorescence microscope (Leica Microsystems, Wetzlar, Germany) at 400× magnification.

### Enzyme-linked immunosorbent assay

The effect of AHWE and AHEE on cytokine production in cisplatin-treated HEK-293 cells was assessed by quantifying cytokine levels in cell-free supernatants using IL-6 and TNF-α ELISA kits (RayBiotech, Norcross, GA, USA). Briefly, 100 μL of each sample was added to the pre-coated wells and incubated at room temperature for 2 h. Following washing steps, biotinylated detection antibodies were applied, and the substrate reaction was initiated. Absorbance was measured at 450 nm using an EZRead 400 microplate reader, and cytokine concentrations were calculated using a standard curve.

### Immunofluorescence staining

Immunofluorescence staining was performed to assess the nuclear translocation of transcription factor NF-κB in the cell nucleus. Following treatment with AHWE and AHEE, cells were washed twice in PBS, fixed with 4% PFA for 30 min at 4°C and incubated overnight with an NF-κB antibody (1:100 dilution, Santa Cruz Biotechnology, Dallas, TX, USA). Following air drying, the slides were incubated with a secondary antibody (1:2000 dilution, Invitrogen, USA) for 60 min. Subsequently, DAPI staining (100 ng/mL, Beyotime, Shanghai, China) was performed at 37°C, followed by three washes with PBS. The slides were then washed twice in PBS, air-dried, treated with a mounting medium, and examined under a fluorescence microscope at 400× magnification. Images were acquired using Leica Application Suite X software.

### Statistical analysis

All experiments were performed in triplicate, and data are presented as the mean ± standard deviation (SD). Group differences were analyzed using one-way ANOVA, followed by Duncan’s multiple range test, using SPSS version 25.0 (SPSS Institute, Chicago, IL, USA). Statistical significance was defined as *P* < 0.05, with specific significance values provided in the figure legends.

## Results

### Effects of AHWE and AHEE on cell viability in cisplatin-treated HEK-293 cells

**Fig. 1:**
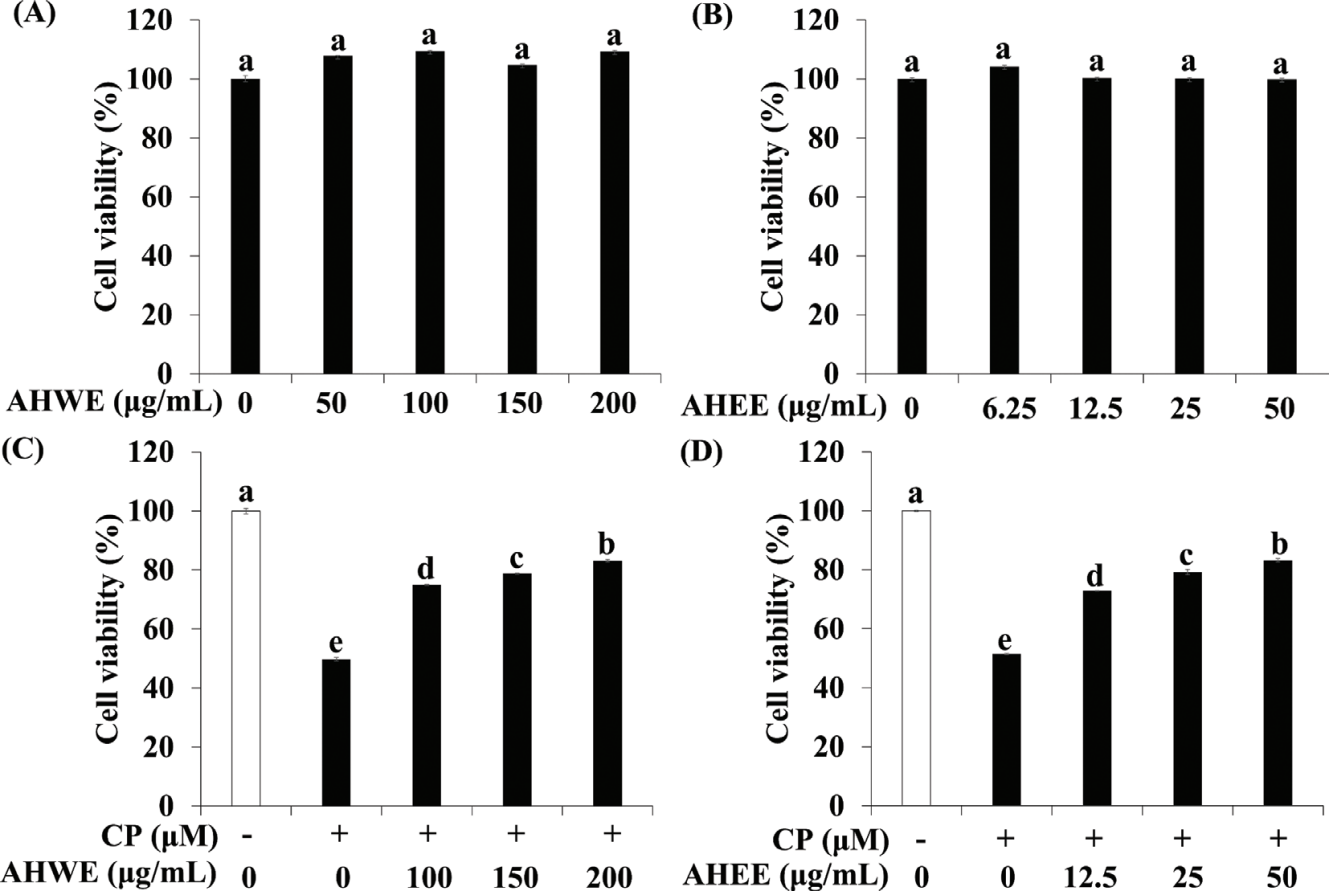
AHWE and AHEE increased cell viability in cisplatin-treated HEK-293 cells. (A-B) Cells were exposed to various concentrations of AHWE (50–200 μM) and AHEE (6.25–50 μM) for 24 h, and cell viability was measured using the MTT assay. (C-D) Cells were pretreated with AHWE and AHEE at several concentrations and then induced with or without 50 μM cisplatin for 24 h. Experiments were conducted at least in triplicate, and results are presented as mean ± SD. Different letters indicate significant differences (*P* < 0.05) according to Duncan’s multiple range test. CP, cisplatin; AHWE, *Allium hookeri* hot water extract; AHEE, *Allium hookeri* ethanol extract; HEK-293 cells, human embryonic kidney-293 cells; MTT, 3-(4,5-Dimethyl-2-thiazolyl)-2,5-diphenyl-2H-tetrazolium Bromide; SD, standard deviation.

The effects of AHWE and AHEE on cell viability were assessed in HEK-293 cells. Neither AHWE nor AHEE exhibited cytotoxicity toward HEK-293 cells for 48 h (Fig. 1A–B). Consequently, non-toxic concentration ranges of AHWE and AHEE were utilized in subsequent experiments. To evaluate the cytotoxic effects of cisplatin, cells were exposed to varying concentrations of the compound. Cisplatin at 50 μM induced the death of approximately 50% of the cells within 24 h, establishing its IC_50_ value at 50 μM. Therefore, a 50 μM concentration of cisplatin was selected for the study. As shown in Fig. 1C–D, AHWE and AHEE treatments significantly improved cell viability in cisplatin-treated HEK-293 cells compared to cisplatin treatment alone. The non-toxic concentrations of AHWE and AHEE, alongside the IC_50_ dosage of cisplatin, were consistently applied in all subsequent experiments.

### Effects of AHWE and AHEE on ROS and NO production in cisplatin-treated HEK-293 cells

**Fig. 2:**
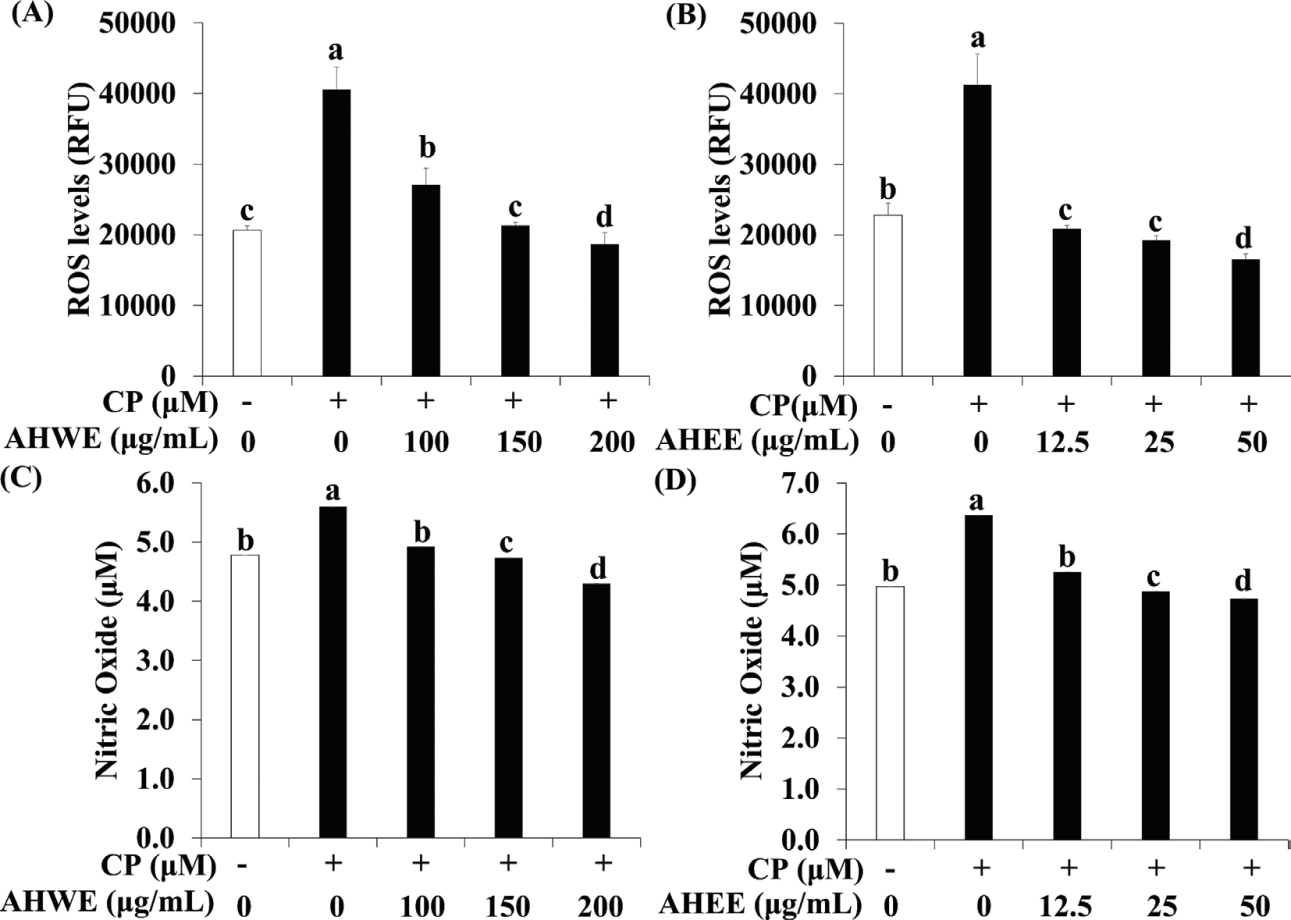
AHWE and AHEE suppressed ROS and NO production in cisplatin-induced HEK-293 cells. (A–B) Intracellular ROS accumulation was assessed using the DCFDA assay with fluorescence measured at Ex/Em = 485/535 nm. (C–D) HEK-293 cells were treated with AHWE and AHEE for 24 h, followed by exposure to cisplatin for 24 h before harvesting. The NO concentration in the culture supernatant was measured using the Griess reagent. Experiments were performed in triplicate, and results are expressed as mean ± SD. Different letters indicate significant differences (*P* < 0.05) according to Duncan’s multiple range test. CP, cisplatin; AHWE, *Allium hookeri* hot water extract; AHEE, *Allium hookeri* ethanol extract; HEK-293 cells, human embryonic kidney-293 cells; ROS, reactive oxygen species; NO, nitric oxide; SD, standard deviation.

The influence of AHWE and AHEE on ROS and NO production in cisplatin-treated HEK-293 cells was evaluated using DCFDA-DA and NO assays. As depicted in Fig. 2A–B, intracellular ROS levels were significantly increased in the cisplatin-treatment group compared to the untreated group. However, treatment with AHWE and AHEE significantly reduced ROS levels. Similarly, cisplatin treatment elevated NO production, while AHWE and AHEE treatments effectively decreased NO levels (Fig. 2C–D). These findings indicate that AHWE and AHEE dose-dependently reduce intracellular ROS and NO production in cisplatin-treated HEK-293 cells.

### Effects of AHWE and AHEE on pro-inflammatory cytokine release and NF-*κ*B pathway-related gene expression in cisplatin-treated HEK-293 cells

**Fig. 3:**
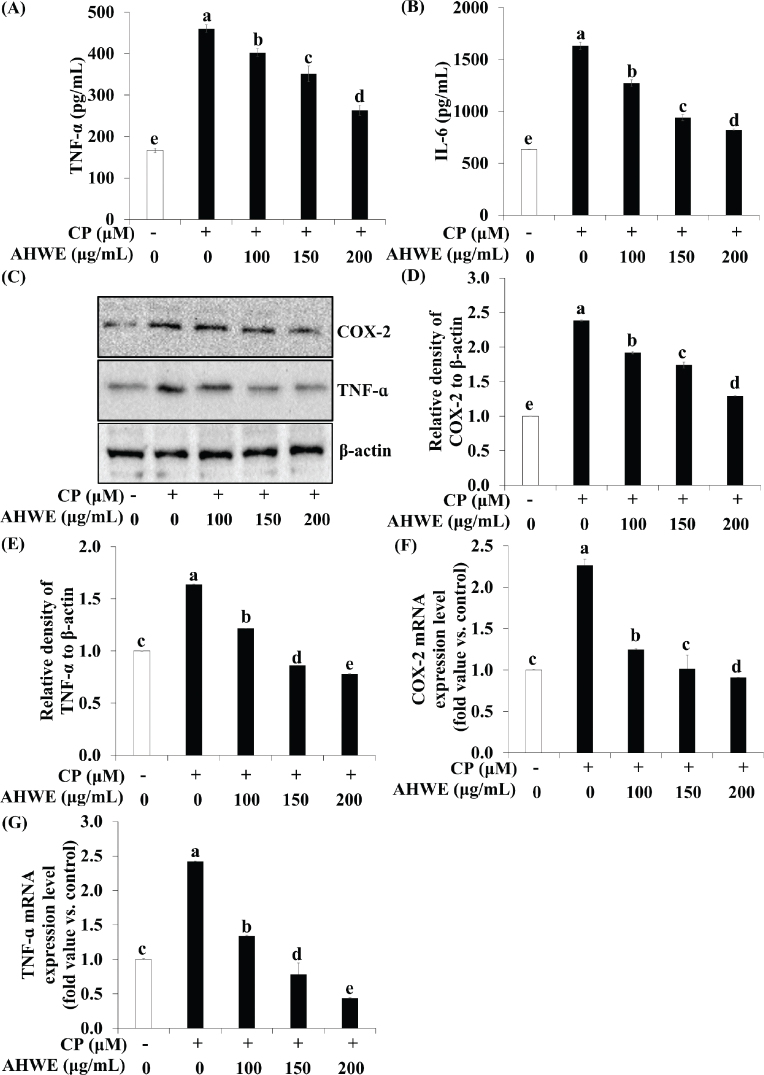
AHWE inhibited the inflammatory cytokine secretion in cisplatin-induced HEK-293 cells. HEK-293 cells were treated with AHWE for 24 h and then exposed to cisplatin for 24 h before harvest. (A-B) The secretion of IL-6 and TNF-ɑ was quantified using an ELISA kit. TNF-ɑ and COX-2 expression levels were assessed using (C) immunoblotting, and (D–E) densities were normalized to β-actin using the ImageJ software. (D) COX-2 and (E) TNF-ɑ levels. Cells were harvested, and the expression levels of (F–G) TNF-ɑ and COX-2 mRNA in cisplatin-induced HEK-293 cells were evaluated. (F) COX-2 and (G) TNF-ɑ levels. Experiments were conducted at least in triplicate, and results are presented as mean ± SD. Different letters indicate significant differences (*P* < 0.05) according to Duncan’s multiple range test. CP, cisplatin; TNF-α, tumor necrosis factor-ɑ; IL-6, interleukin-6; COX-2, cyclooxygenase-2; AHWE, *Allium hookeri* hot water extract; HEK-293 cells, human embryonic kidney-293 cells; ELISA, enzyme-linked immunosorbent assay; SD, standard deviation.

**Fig. 4:**
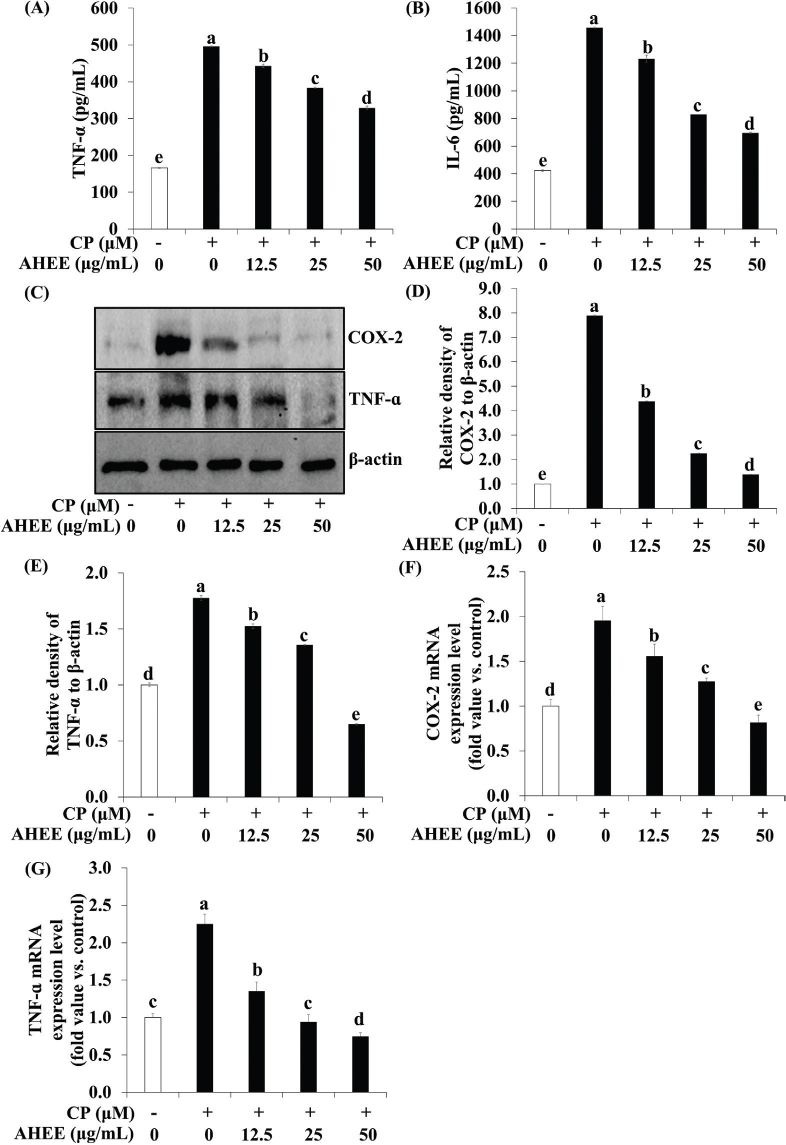
AHEE inhibited the inflammatory cytokine secretion in cisplatin-induced HEK-293 cells. HEK-293 cells were treated with AHEE for 24 h and then exposed to cisplatin for 24 h before harvest. (A-B) The secretion of IL-6 and TNF-α was quantified using an ELISA kit. TNF-ɑ and COX-2 expression levels were assessed using (C) immunoblotting, and (D–E) densities were normalized to β-actin using the ImageJ software. (D) COX-2 and (E) TNF-ɑ levels. Cells were harvested, and the expression of (F–G) TNF-ɑ and COX-2 mRNA in cisplatin-induced HEK-293 cells was evaluated. (F) COX-2 and (G) TNF-ɑ levels. Experiments were conducted at least in triplicate, and the results are presented as mean ± SD. Different letters indicate significant differences (*P* < 0.05) according to Duncan’s multiple range test. CP, cisplatin; TNF-α, tumor necrosis factor-ɑ; IL-6, interleukin-6; COX-2, cyclooxygenase-2; AHEE, *Allium hookeri* ethanol extract; HEK-293 cells, human embryonic kidney-293 cells; ELISA, enzyme-linked immunosorbent assay; SD, standard deviation.

**Fig. 5:**
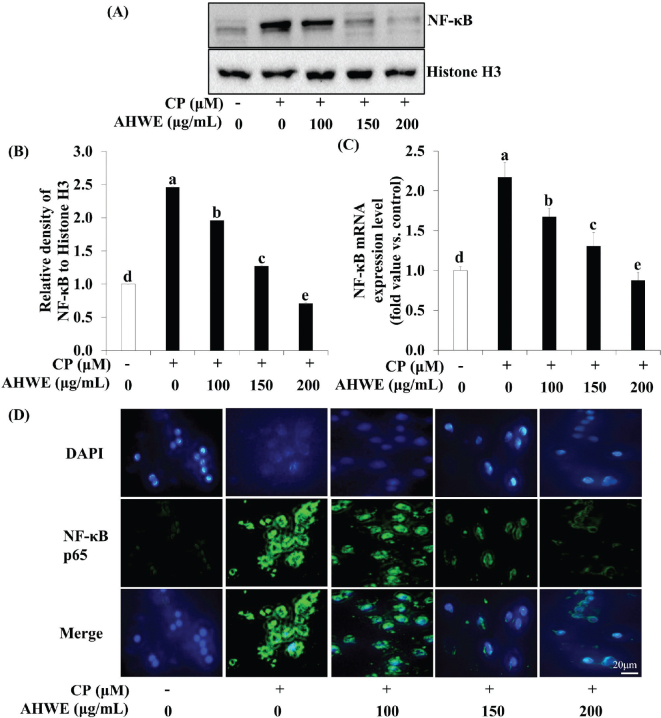
Effects of AHWE on NF-κB activation in cisplatin-treated HEK-293 cells. NF-κB protein levels were assessed using (A) immunoblotting, and (B) densities were normalized to β-actin using the ImageJ software. Cells were harvested, and the expression of (C) NF-κB mRNA in cisplatin-induced HEK-293 cells was evaluated. Data are presented as the means ± SD. Different letters indicate significant differences (*P* < 0.05) according to Duncan’s multiple range test. (D) HEK-293 cells were treated with AHWE and then fixed with 4% paraformaldehyde. Following blocking with an appropriate buffer, cells were incubated with antibodies. Subsequently, DAPI staining was performed to visualize cell nuclei, and signals were quantified using fluorescence microscopy at 400× magnification. CP, cisplatin; TNF-α, tumor necrosis factor-ɑ; IL-6, interleukin-6; COX-2, cyclooxygenase-2; AHWE, *Allium hookeri* hot water extract; HEK-293 cells, human embryonic kidney-293 cells; NF-κB, nuclear factor-κB; DAPI, 4′,6-diamidino-2-phenylindole; SD, standard deviation.

**Fig. 6:**
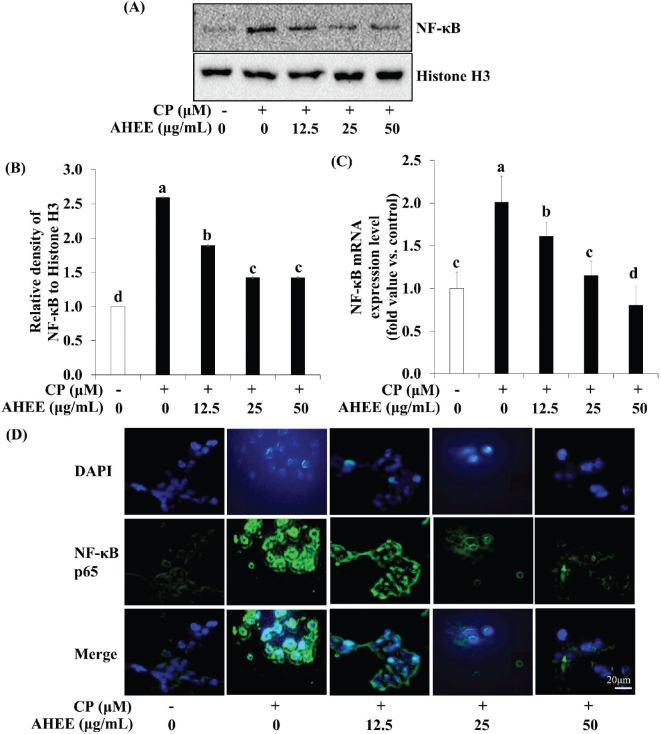
Effects of AHEE on NF-κB activation in cisplatin-treated HEK-293 cells. NF-κB protein levels were assessed using (A) immunoblotting, and (B) densities were normalized to β-actin using the ImageJ software. Cells were harvested, and the expression of the (C) NF-κB mRNA in cisplatin-induced HEK-293 cells was evaluated. Data are presented as mean ± SD. Different letters indicate significant differences (*P* < 0.05), according to the Duncan’s multiple range test. (D) HEK-293 cells were treated with AHEE, fixed with 4% paraformaldehyde, and then incubated with antibodies after blocking. Subsequently, DAPI staining confirmed the presence of nuclei, and signals were quantified using fluorescence microscopy at 400× magnification. CP, cisplatin; TNF-α, tumor necrosis factor-ɑ; IL-6, interleukin-6; COX-2, cyclooxygenase-2; AHEE, *Allium hookeri* ethanol extract; HEK-293 cells, human embryonic kidney-293 cells; NF-κB, nuclear factor-κB; DAPI, 4′,6-diamidino-2-phenylindole; SD, standard deviation.

The impact of AHWE and AHEE on pro-inflammatory cytokine secretion and NF-κB pathway-related gene expression in cisplatin-induced nephrotoxicity was examined. ELISA assays demonstrated a significant upregulation of inflammatory cytokines TNF-α and IL-6 in cisplatin-induced nephrotoxicity (Figs. 3A–B and 4A–B). However, treatment with AHWE and AHEE significantly inhibited this cytokine overproduction (*P* < 0.05). Furthermore, the expression of COX-2 and TNF-α proteins was elevated in cisplatin-treated HEK-293 cells, while AHWE and AHEE treatments markedly reduced their expression (Fig. 3C and 4C). Excessive ROS production induced by cisplatin is known to influence the expression of the NF-κB gene, a critical factor in the NF-κB-mediated renal disease pathway (31). AHWE and AHEE treatments significantly decreased NF-κB protein levels (*P* < 0.05) and reduced mRNA expression of the NF-κB gene compared to the cisplatin control group (*P* < 0.05) (Fig. 5A–C and 6A–C). Immunofluorescence analysis further confirmed that AHWE and AHEE inhibited cisplatin-induced p65 nuclear translocation (Fig. 5D and 6D). These results suggest that AHWE and AHEE possess therapeutic potential as inhibitors of cisplatin-induced renal inflammation.

### Effects of AHWE and AHEE on apoptosis signaling in cisplatin-treated HEK-293 cells

**Fig. 7:**
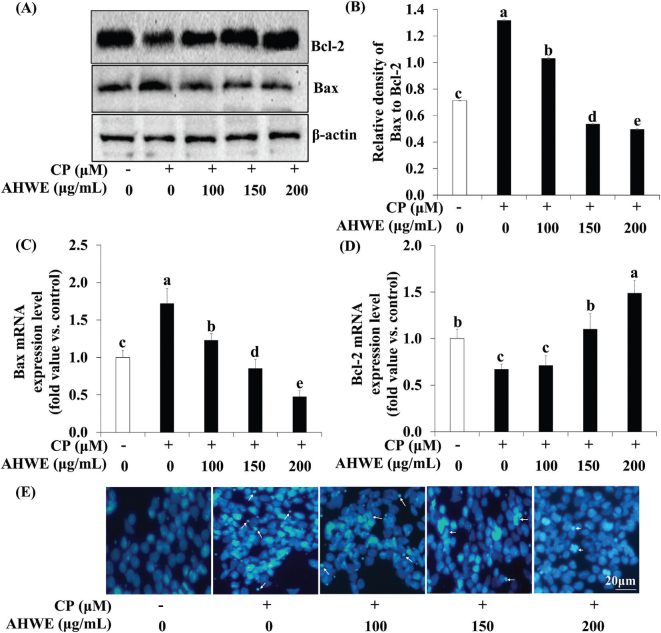
AHWE inhibited cisplatin-induced apoptosis in cisplatin-treated HEK-293 cells. (A) Immunoblotting was utilized to measure protein expression levels of Bax and Bcl-2. (B) ImageJ software was employed to quantify Bax/Bcl-2 levels. (C–D) Relative mRNA expression levels are shown after normalization against β-actin mRNA expression. (C) Bax and (D) Bcl-2 levels. Data are expressed relative to mRNA levels in untreated cells, which was arbitrarily defined as 1. Experiments were conducted at least in triplicate, and the results are expressed as mean ± SD. Data analysis was performed using the 2^-ΔΔCT^ method, with different letters indicating significant differences (*P* < 0.05) as determined through the Duncan’s multiple range test. (E) HEK-293 cells were treated with AHWE for 24 h, fixed with 4% paraformaldehyde, and signal quantification was assessed using a fluorescence microscope at 400× magnification. CP, cisplatin; Bax, Bcl-2-associated X protein; Bcl-2, B-cell lymphoma 2; AHWE, *Allium hookeri* hot water extract; HEK-293 cells, human embryonic kidney-293 cells; SD, standard deviation.

**Fig. 8:**
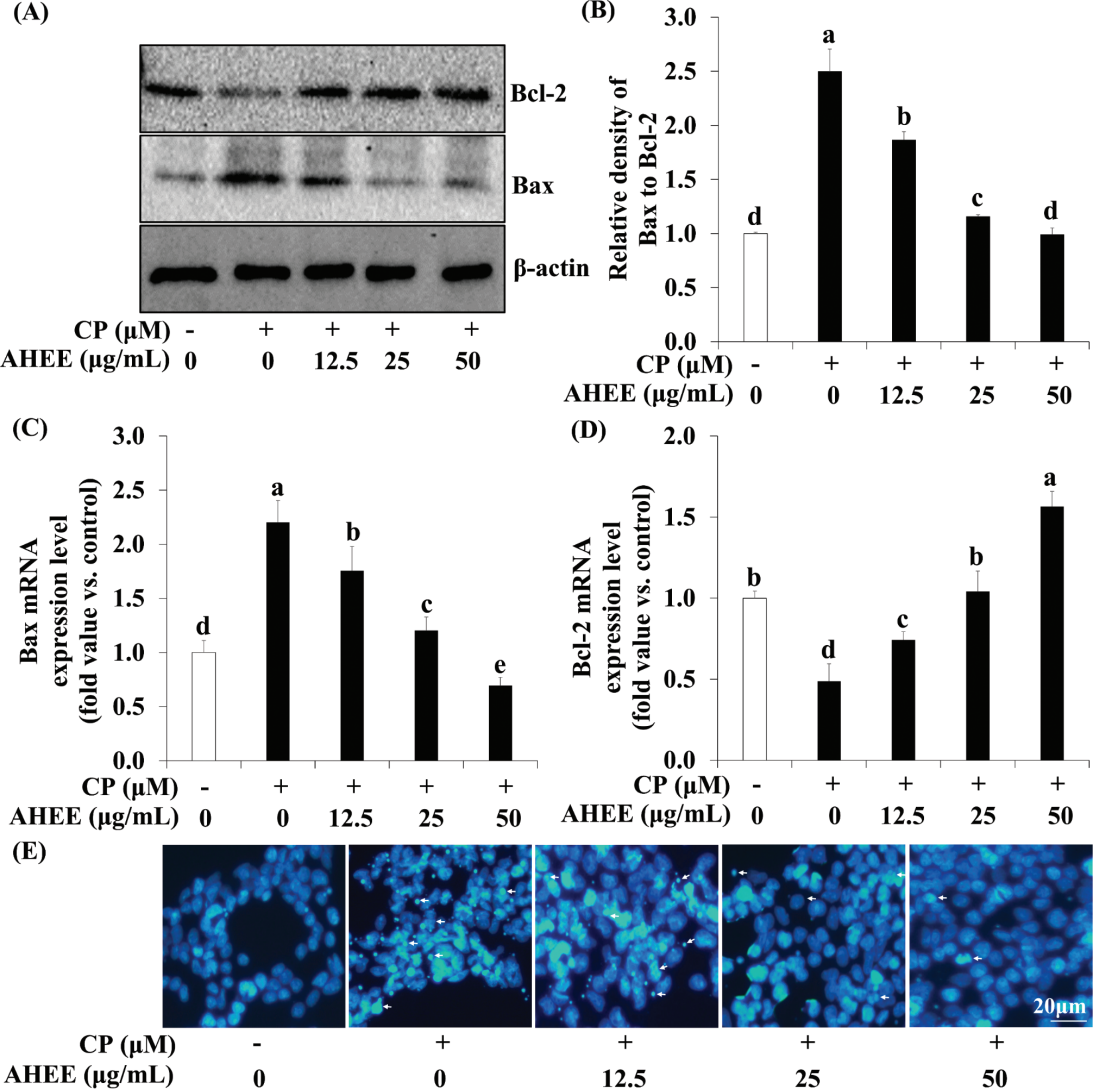
AHEE inhibited cisplatin-induced apoptosis in cisplatin-treated HEK-293 cells. (A) Immunoblotting was utilized to measure protein expression levels of Bax and Bcl-2. (B) ImageJ software was employed to quantify Bax/Bcl-2 levels. (C–D) The relative mRNA expression levels are shown following normalization against β-actin mRNA expression. (C) Bax and (D) Bcl-2 levels. Data are expressed relative to mRNA levels in untreated cells, defined as 1. Experiments were conducted at least in triplicate, and the results are expressed as mean ± SD. Data analysis was performed using the 2^-ΔΔCT^ method, with different letters indicating significant differences (*P* < 0.05) as determined by the Duncan’s multiple range test. (E) HEK-293 cells were treated with AHEE for 24 h, fixed with 4% paraformaldehyde, and signal quantification was assessed using a fluorescence microscope at 400× magnification. CP, cisplatin; Bax, Bcl-2-associated X protein; Bcl-2, B-cell lymphoma 2; AHEE, *Allium hookeri* ethanol extract; HEK-293 cells, human embryonic kidney-293 cells; SD, standard deviation.

To investigate whether AHWE and AHEE modulate apoptosis signaling and reduce cell death in cisplatin-treated HEK-293 cells, we analyzed Bax and Bcl-2 expression through immunoblotting and qPCR. Cisplatin treatment led to an increase in the expression of the proapoptotic factor Bax compared to untreated cells. However, treatment with AHWE and AHEE reduced Bax expression in cisplatin-treated HEK-293 cells. Additionally, AHWE and AHEE exposure resulted in significantly increased expression of the antiapoptotic protein Bcl-2 compared to the cisplatin-treated group (Fig. 7A and 8A). The Bax/Bcl-2 ratio was significantly lower in the AHWE and AHEE treatment groups than in the cisplatin-treated group (*P* < 0.05) (Fig. 7B and 8B). At the mRNA level, *Bax* expression was elevated, and *Bcl-2* expression was decreased in cisplatin-treated cells relative to untreated controls. However, treatment with AHWE and AHEE significantly decreased Bax mRNA levels and markedly increased Bcl-2 mRNA levels (*P* < 0.05) (Fig. 7C–D and 8C–D). Hoechst 33342 staining further confirmed the effects of AHWE and AHEE on nuclear morphology in cisplatin-treated HEK-293 cells. As shown in Figs. 7E and 8E, cisplatin treatment induced apoptotic bodies and nuclear condensation, while AHWE and AHEE treatment inhibited these effects in HEK-293 cells exposed to 50 μM cisplatin (*P* < 0.05).

### Effects of AHWE and AHEE on MAPK activation in cisplatin-treated HEK-293 cells

**Fig. 9:**
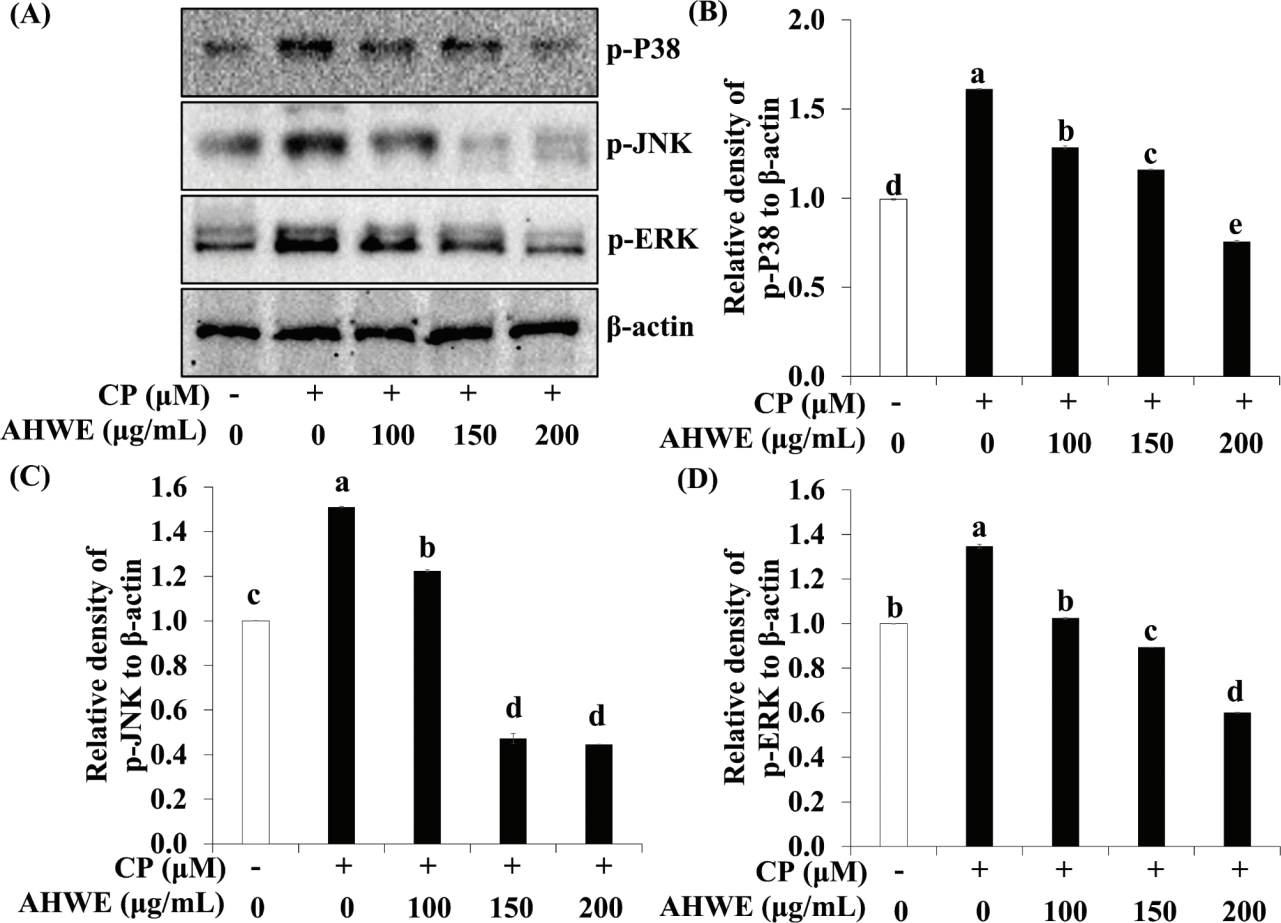
Effects of AHWE in the MAPK signaling pathway in cisplatin-treated HEK-293 cells. (A) Immunoblotting was employed to measure the expression levels of p-P38, p-JNK, and p-ERK, while (B–D) ImageJ software was utilized to normalize their densities to β-actin. (B) p-P38, (C) p-JNK, and (D) p-ERK levels. Experiments were performed at least in triplicate, and results are presented as mean ± SD. Different letters denote significant differences (*P* < 0.05) as determined via the Duncan’s multiple range test. CP, cisplatin; MAPK, mitogen-activated protein kinase; AHWE, *Allium hookeri* hot water extract; p-, phosphorylated; ERK, extracellular signal-regulated kinase; JNK, c-Jun N-terminal kinase; p38, p38 mitogen-activated protein kinases; HEK-293 cells, human embryonic kidney-293 cells; SD, standard deviation.

**Fig. 10:**
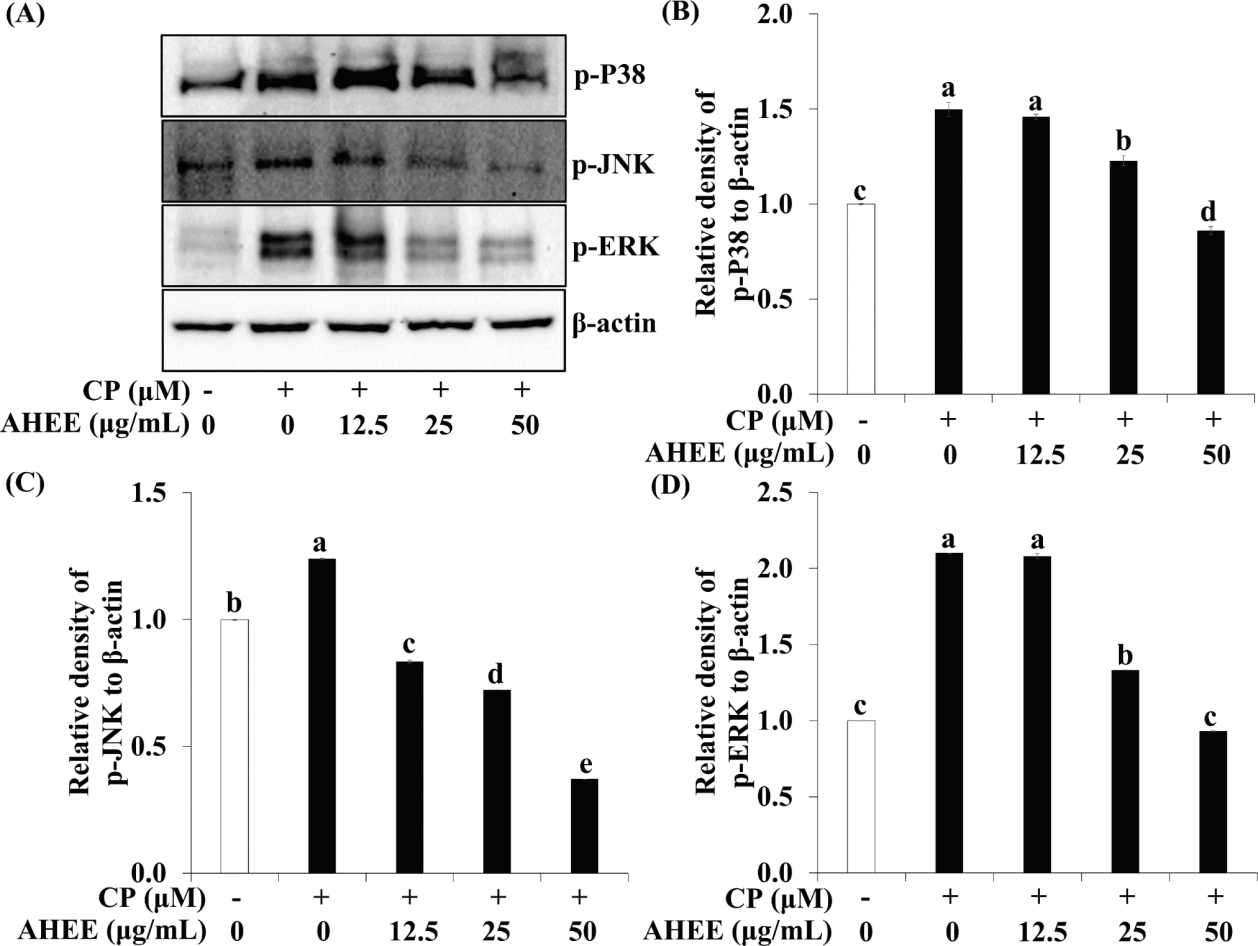
Effects of AHEE in the MAPK signaling pathway in cisplatin-treated HEK-293 cells. (A) Immunoblotting was utilized to assess the expression levels of p-P38, p-JNK, and p-ERK. (B–D) ImageJ software was employed to normalize their densities to β-actin. (B) p-P38, (C) p-JNK, and (D) p-ERK levels. Experiments were conducted in triplicate, and results are presented as mean ± SD. Statistical significance (*P* < 0.05) was determined using the Duncan’s multiple range test with different letters indicating significant differences. CP, cisplatin; MAPK, mitogen-activated protein kinase; AHEE, *Allium hookeri* ethanol extract; p-, phosphorylated; ERK, extracellular signal-regulated kinase; JNK, c-Jun N-terminal kinase; p38, p38 mitogen-activated protein kinases; HEK-293 cells, human embryonic kidney-293 cells; SD, standard deviation.

Since MAPK pathway activation is closely associated with renal cell apoptosis (32), we evaluated the impact of AHWE and AHEE on MAPK signaling in cisplatin-treated HEK-293 cells. As depicted in Figs. 9 and 10, cisplatin treatment increased the phosphorylation of ERK, JNK, and p38 kinase, indicating MAPK pathway activation. In contrast, AHWE and AHEE treatment inhibited the phosphorylation of p-ERK, p-JNK, and p-P38. These findings suggest that AHWE and AHEE may mitigate cisplatin-induced cell death by suppressing MAPK pathway activation.

## Discussion

The platinum-based chemotherapy agent, cisplatin, is widely utilized in the treatment of various cancers (8). As a first-line therapy for solid tumors, cisplatin forms covalent bonds with the DNA of rapidly dividing cancer cells, thereby inhibiting DNA replication and transcription, ultimately suppressing cancer cell proliferation. Additionally, cisplatin is effective in activating anticancer immune responses and inhibiting intratumoral vascular endothelial growth factor signaling, thereby preventing angiogenesis (33). Despite its clinical efficacy, the therapeutic use of cisplatin is limited by its adverse effects, particularly nephrotoxicity. Cisplatin predominantly accumulates in renal tissue, inducing nephrotoxicity through the loss of renal endothelial cells (34, 35). Specifically, cisplatin triggers apoptotic pathways and induces cellular damage via oxidative stress and inflammation, culminating in acute renal failure (36). The pathophysiology of cisplatin-induced acute renal failure involves a multifaceted process, with renal cell death and inflammation as key pathological features (33). Elevated levels of inflammatory cytokines activate the MAPK and NF-κB pathways, promoting apoptosis and leading to renal dysfunction (17). Currently, no clinically effective pharmacological interventions exist to prevent, reduce, or mitigate cisplatin-induced nephrotoxicity. Many recent studies have explored the potential of safe natural products with known physiological activities as dietary supplements, clinical combination therapies, and intravenous treatments for cisplatin-induced nephrotoxicity (37). Notable examples include research on natural dietary nephroprotective compounds such as resveratrol, apigenin, and emodin (37–39). *A. hookeri*, a plant native to East Asia, is rich in proteins, phytosterols, and dietary sulfur and demonstrates anti-inflammatory, antioxidant, and antitumor properties (40–42). To date, the nephroprotective effects of *A. hookeri* and its underlying molecular mechanisms have not been elucidated. Therefore, this study aimed to investigate the nephroprotective effects of AHWE and AHEE on cisplatin-induced nephrotoxicity in HEK-293 cells.

ROS, which are crucial byproducts of cellular metabolic processes, are typically regulated by the cellular antioxidant system (37). However, dysregulation of this balance leads to excessive ROS production, causing damage to cellular lipid membranes, intracellular proteins, and DNA (38). In renal cells exposed to cisplatin, increased intracellular ROS production accelerates cell apoptosis (39). The excessive accumulation of ROS generated by cisplatin is a hallmark of nephrotoxicity.

ROS generated by cisplatin are involved in a pathway that interacts with surrounding compounds and infiltrates kidney tissue, contributing to nephrotoxicity (40). Elevated ROS levels activate the MAPK signaling pathway and disrupt mitochondrial membrane potential, leading to apoptosis and inflammation. NO, a regulator of ROS, produces cytotoxic peroxynitrite, significantly contributing to cell death and oxidative stress (41). Previous studies have demonstrated the potential of natural compounds to mitigate ROS production. Yu et al. reported that ruscogenin (2.5–10 μM) effectively reduced ROS levels in cisplatin-induced renal tubular epithelial cells (42). Similarly, Tusskorn et al. found that Borassus flabellifer L. crude male flower extracts (1–10 μg/mL) decreased ROS levels in cisplatin-induced rat kidney cells (43). Our study corroborates these findings, demonstrating that pretreatment with AHWE (100–200 μg/mL) and AHEE (12.5–50 μg/mL) significantly reduced ROS and NO production in cisplatin-treated HEK-293 cells. Consistent with our results, a dose-dependent decrease in cell viability was observed in cisplatin-treated HEK-293 cells. However, pretreatment with AHWE and AHEE significantly enhanced cell viability, underscoring the renal protective effects of these extracts against cisplatin-induced toxicity in HEK-293 cells.

Apoptosis, a physiological process governed by multiple cell death signaling pathways, is commonly triggered by diverse pathological stimuli (44). Elevated apoptosis has been documented in the kidneys of patients with kidney disease (45). Apoptotic cells exhibit distinct morphological changes, including cell contraction, rounding, and the formation of apoptotic bodies. Hoechst 33342 staining in our study revealed that AHWE and AHEE marginally attenuated cisplatin-induced apoptotic characteristics compared to cisplatin treatment alone. This staining technique specifically assesses apoptosis by evaluating changes in nuclear morphology, such as cell condensation and fragmentation, in cisplatin-treated HEK-293 cells (46). Supporting these findings, Fan et al. reported that daphnetin (2.5–10 μg/mL) inhibited nuclear morphological changes, including cell nuclear condensation, in cisplatin-treated HK-2 cells (47). The Bcl-2 family of proteins plays a critical role in regulating apoptosis. Bcl-2 is a key antiapoptotic protein that promotes cell survival, while Bax is a proapoptotic protein that translocates to the mitochondria early in apoptosis, facilitating cell death (48). The Bax/Bcl-2 ratio is a crucial indicator of caspase-3 activity and apoptosis (49). Chen et al. demonstrated that hesperetin (2.5–10 μM) inhibited apoptosis by modulating the expression of Bax and Bcl-2 in cisplatin-induced HK-2 cells (50). In alignment with these findings, our study showed that treatment with AHWE (100–200 μg/mL) and AHEE (12.5–50 μg/mL) upregulated Bcl-2 and downregulated Bax expression in cisplatin-treated HEK-293 cells. Moreover, AHWE and AHEE treatment effectively reduced the increased Bax/Bcl-2 ratio.

Excessive accumulation of ROS induced by cisplatin, as previously described, activates multiple signaling pathways, including MAPK, leading to damage to cellular macromolecules, such as lipids, proteins, and DNA (51). The MAPK signaling cascade, which governs critical biological responses such as cell proliferation and apoptosis, is initiated by the activation of cell membrane receptors through extracellular stimuli (52). Evidence suggests that MAPK activation following cisplatin exposure impairs renal function and promotes apoptosis and inflammation (53, 54). Specifically, p38 activation enhances intracellular ROS levels and increases TNF-α production (55). In kidney cells, ERK activation triggers Bax activation, decreases Bcl-2 expression, facilitates Bax translocation from the cytoplasm to the mitochondria, promotes cytochrome c release into the cytoplasm, and induces apoptosis (56). The apoptosis induced by cisplatin involves complex formation between JNK and proapoptotic components of p53, contributing to cell death regulation (57). Diomede et al. demonstrated that oleuropein attenuated the phosphorylation of MAPK proteins in LPS-treated HEK-293 cells (58). Similarly, our results demonstrate that pretreatment with AHWE and AHEE inhibited ERK, JNK, and p38 activation in cisplatin-treated HEK-293 cells.

Inflammation is a critical contributor to the progression of cisplatin-induced renal damage (59). The transcription factor NF-κB, a key regulator of inflammatory responses, enhances the expression of pro-inflammatory mediators, including TNF-α, IL-12, and iNOS, leading to excessive NO production (31, 60). Cisplatin-mediated phosphorylation of IKKɑ/β triggers ubiquitination and proteasomal degradation of IκB, facilitating the release and nuclear translocation of NF-κB p65, thereby exacerbating renal injury (61). TNF-α is particularly pivotal in cisplatin-induced nephrotoxicity (62). Our findings reveal that cisplatin exposure upregulates the protein expression of NF-κB, TNF-α, and COX-2 in HEK-293 cells, signifying the activation of inflammatory pathways. In contrast, treatment with AHWE and AHEE effectively reduced the expression of TNF-α, COX-2, and NF-κB. Supporting this observation, Yu et al. reported that ruscogenin mitigates inflammation in cisplatin-treated renal epithelial cells by inhibiting TLR4/NF-κB signaling (42), while Pei et al. showed that isoliquirtin decreases IL-6 and TNF-α levels in cisplatin-treated HK2 cells (63). These findings collectively indicate that AHWE and AHEE attenuate cisplatin-induced renal inflammation by modulating NF-κB expression. Furthermore, AHWE and AHEE appear to inhibit apoptosis by regulating Bax and Bcl-2 in the context of cisplatin-induced nephrotoxicity. This regulatory effect is likely mediated through the suppression of ROS, including ROS and NO, within cells. The overall nephroprotective mechanism of AHWE and AHEE may, therefore, involve the inactivation of MAPK and NF-κB signaling pathways, highlighting a central regulatory mechanism in mitigating cisplatin-induced kidney damage.

## Conclusions

This study demonstrates that AHWE and AHEE inhibit apoptosis by modulating the MAPK and NF-κB signaling pathways. Specifically, treatment with AHWE and AHEE reduced the expression of inflammation-related genes (TNF-α and IL-6) and suppressed the expression of MAPK and NF-κB target genes associated with inflammation, cell stress, apoptosis, and cell growth in cisplatin-treated HEK-293 cells. Collectively, these findings indicate that AHWE and AHEE confer renal protective effects under the conditions of this study and highlight their potential as natural therapeutic agents against nephrotoxicity. The anti-inflammatory and antioxidant properties of *A. hookeri* extract suggest promising clinical applications in mitigating cisplatin-induced nephrotoxicity. Given that *A. hookeri* is already widely used as a food source, its development as a dietary supplement or as a component of clinical combination therapies – using active single compounds isolated from *A. hookeri* – could provide a novel approach for managing cisplatin-induced nephrotoxicity. Future studies will focus on elucidating the precise molecular mechanisms by which these active compounds ameliorate nephrotoxic effects.
